# Use of vouchers for CPAP therapy initiation - public and private health care could work together

**DOI:** 10.1007/s11325-024-03159-1

**Published:** 2024-09-20

**Authors:** Toni Jämsänen, Pirkko Brander, Adel Bachour

**Affiliations:** Sleep Unit, Heart and Lung Center, Helsinki University Hospital, Helsinki, Finland

**Keywords:** CPAP, Sleep apnea, Private sector, Voucher

## Abstract

**Purpose:**

The increasing incidence of sleep apnea has led to an increased workload for healthcare professionals. Continuous positive airway pressure (CPAP) is the gold standard therapy for obstructive sleep apnea. To reduce the CPAP waiting list in public healthcare, we proposed a CPAP voucher for use in private clinics for CPAP initiation. This study evaluated the success rate of CPAP initiation via this voucher.

**Methods:**

We selected patients from our sleep apnea clinic referred to CPAP initiation aged 18–80 years with no significant physical or psychological comorbidity. Three private clinics (A, B, C) accepted the CPAP voucher.

**Results:**

A total of 1922 patients fulfilled CPAP voucher criteria. Of these, we included 1604 patients (38% women). Mean BMI was 32 kg/m^2^, mean age was 55 years, and mean apnea-hypopnea index was 34/h. Data were missing for 113 patients at the 1-year follow-up visit. Of the remaining 1491 patients, 1398 continued CPAP therapy beyond 1 year, indicating a CPAP therapy success rate of 94%. There were no significant differences between clinics in the number of patients staying on CPAP at 1 year after initiation.

**Conclusion:**

A CPAP voucher may reduce the CPAP initiation waiting list in public healthcare with a good success rate.

## Introduction

Obstructive sleep apnea (OSA) is a common disorder that is associated with increased daytime sleepiness [1], cardiovascular disease [2], and reduced quality of life [3]. Worldwide, the disease burden is estimated to affect almost a billion people [4]. In Finland, the prevalence is estimated to be approximately 3.7% and the incidence approximately 0.6% [5]. Continuous positive airway pressure (CPAP) is the first-line therapy and the most effective treatment for moderate and severe sleep apnea. CPAP delivers pressurized air to the upper airways, splinting them open and increasing end expiratory lung volume [6]. Clinical improvement during CPAP is directly related to a major reduction in respiratory events during sleep.

The incidence of sleep apnea is increasing [7], leading to an increased workload for healthcare professionals. Managing this increased workload is challenging with restricted medical budgets. Most governments have difficulties in reducing healthcare costs.

There are several approaches to address the limited resources of public healthcare such as pay-per-act or an agreement with public healthcare and private doctors in which the latter manage a certain number of patients at a previously fixed price or a voucher-based approach, where a public clinic refers a patient to a private clinic to perform a pre-agreed act at a pre-agreed price.

We began use of vouchers for CPAP therapy initiation in May 2020. The purpose of this study was to evaluate the success of CPAP therapy via voucher use.

## Methods

The study period was between 6 May 2020 to 10 October 2023. Patients were referred to our public sleep apnea clinic for sleep apnea therapy. Due to limited human resources, we forwarded selected patients to the private sector for CPAP therapy initiation using a voucher.

### Criteria for voucher use

Patients were required to fulfill all the following criteria for voucher use:

1. Apnea and hypopnea index (AHI) ≥ 15/h, mean sleep study oxygen saturation SpO_2_ > 90%, and upright awake SpO_2_ > 92%.
2. Sleep apnea should be obstructive (apnea/hypopnea > 50% of all respiratory events).
3. Age between 18 and 80 years.
4. No confirmed pregnancy.
5. Patient was not a professional driver or in assimilated profession.
6. Absence of severe comorbidity.
7. Absence of moderate-to-severe impaired cognitive function.
8. Good communication ability.
9. Absence of obesity hypoventilation syndrome.
10. In-hospital CPAP initiation not required.
11. No previous CPAP trial.
12. No contraindications for CPAP therapy.

The voucher covered the following:

1. Face-to-face visit with a sleep nurse specialized in CPAP therapy initiation.
2. Suitable CPAP interface.
3. Leasing of the CPAP device for maximum period of 3 months.
4. Final evaluation of CPAP success by a nurse specialized in sleep medicine.

The value of the voucher was estimated to be approximately 600 US dollars.

Private clinics were invited to participate in voucher CPAP therapy initiation. Every private clinic was required to fulfill all the following criteria before being accepted as an agreed voucher clinic:

1. Experience in sleep apnea therapy.
2. A physician specialized in sleep medicine should be available in case a nurse specialized in sleep medicine requires assistance.

Three clinics fulfilled these criteria. We classified the three private clinics as clinic A, clinic B, and clinic C. The patient had the right to refuse the CPAP voucher; in this case CPAP initiation was performed at our public clinic. The patient could choose between the three private clinics.

The maximal duration of CPAP therapy at the private clinic was 3 months and the minimum period was 3 weeks.

The final report included information about mask type, date of initiation, unintentional leak, CPAP-AHI values, days of CPAP use, mean hours per day, BMI, and any special remarks regarding CPAP initiation.

Patients who were willing to continue CPAP therapy were invited to the public sleep clinic for a new evaluation approximately 3 months after CPAP initiation. The next routine follow up was planned at 1 year after CPAP initiation. An additional visit was planned if necessary.

The AirView TM program was activated in all CPAP devices, which allowed for transfer of CPAP data to the hospital computer. Patients were allowed to inactivate the program.

The major outcome was staying on CPAP 1 year after initiation.

## Statistical analyses

All statistical analyses were performed using IBM SPSS Statistics version 29.0 (SPSS Inc., Chicago, IL, USA). All data are expressed either as means with standard deviation or as absolute numbers with percentages. All analyzed variables were tested for normal distribution. The *t*-test and analysis of variance were used for variables with normal distribution. Mann-Whitney *U*-test was used for parameters with skewed distribution. χ^2^ was used for non-continuous variables. A p-value < 0.05 was considered significant.

## Results

**Fig. 1:**
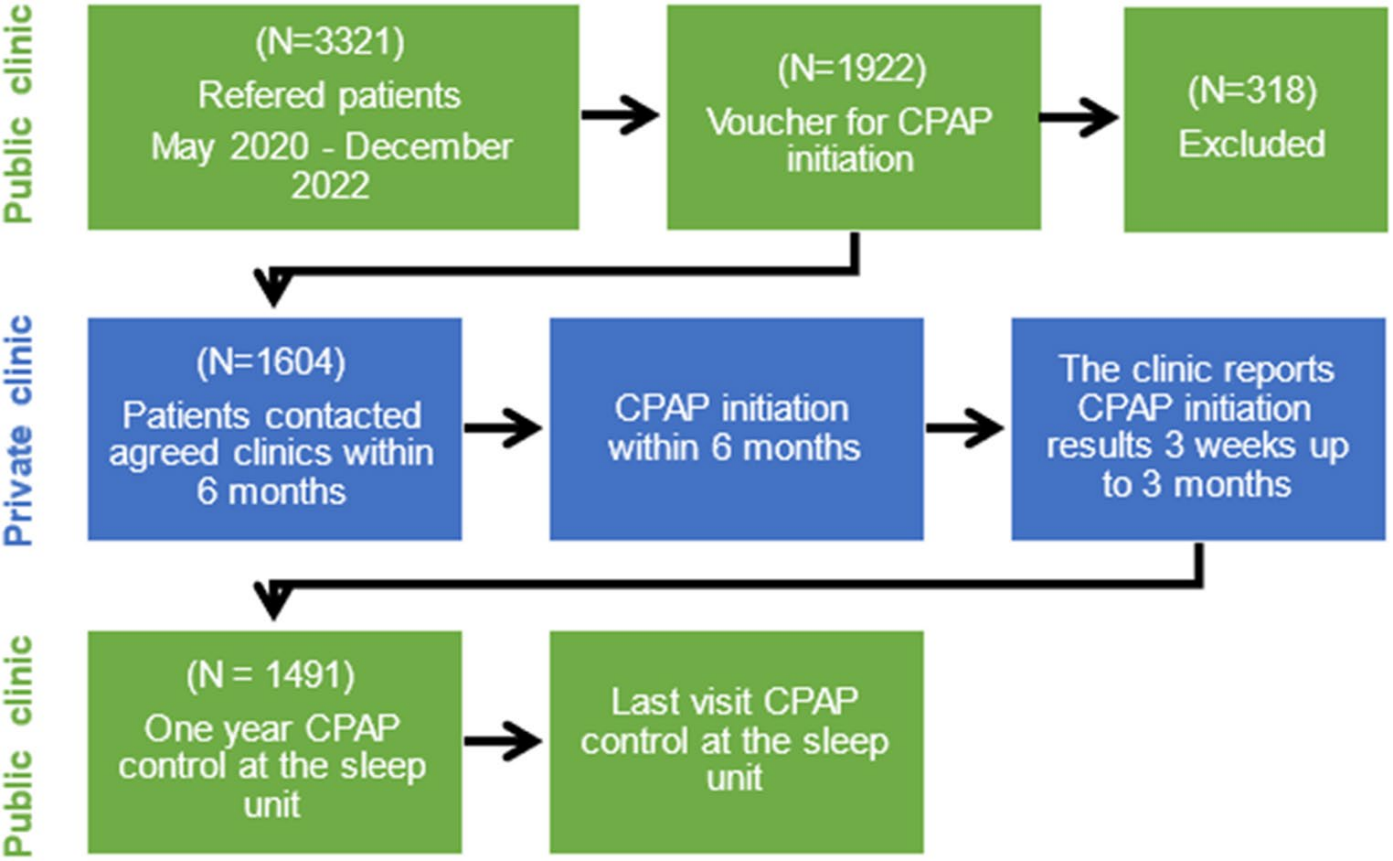
Study flowchart

We received 3321 referred patients for CPAP initiation during the study period; 1922 patients fulfilled the voucher criteria (Fig. 1).

A total of 318 patients did not use their voucher. Most of these patients refused the private clinic voucher, stated that they did not receive the voucher by mail, or postponed CPAP therapy initiation. We included the remaining 1604 patients; 606 (38%) were women. Mean (SD) age was 55 (12) years, mean BMI was 32 (7) kg/m^2^, and mean was AHI 34/h (18). Women were significantly more obese than men (mean [SD] BMI 33.8 [6.8] vs. 31.4 [6.1] kg/m^2^; *p* < 0.001), significantly older (mean [SD] age 57.5 [11.8] vs. 53.6 [12.2] years; *p* < 0.001), and had less severe sleep apnea (mean [SD] 32/h [17] vs. 36/h [19]; *p* = 0.003).

The number of patients treated was 420 (clinic A), 1021 (clinic B), and 163 (clinic C). There were no significant differences between the clinics regarding gender, BMI, age, and sleep apnea severity (Table 1).

**Table 1:** Patient characteristics of different clinics

		Clinics			
		A	B	C	p
Women	N	155	382	69	
	%	36.9%	37.4%	42.3%	0.442
Age	Mean	54.2	55.3	55.7	0.203
BMI	Mean	32.6	32.2	32.5	0.779
AHI	Mean	35.1	34.5	31.7	0.211
Number	N	420	1021	163	
	% of Total	26.2%	63.7%	10.2%	

BMI: Body mass index; AHI: Apnea-hypopnea index

The mean (SD) CPAP follow-up period was 666 (185) days; the maximum and minimum period was 1071 days and 44 days, respectively.

### CPAP outcomes

Data from 113 patients were missing at the 1-year follow-up visit. The reasons for missing data were moving to another district, death, inactivation of AirView program, or temporary pause in CPAP without abandoning. Of the remaining 1491 patients, 1398 continued CPAP therapy beyond 1 year, indicating a CPAP therapy success rate of 94%.

**Fig. 2:**
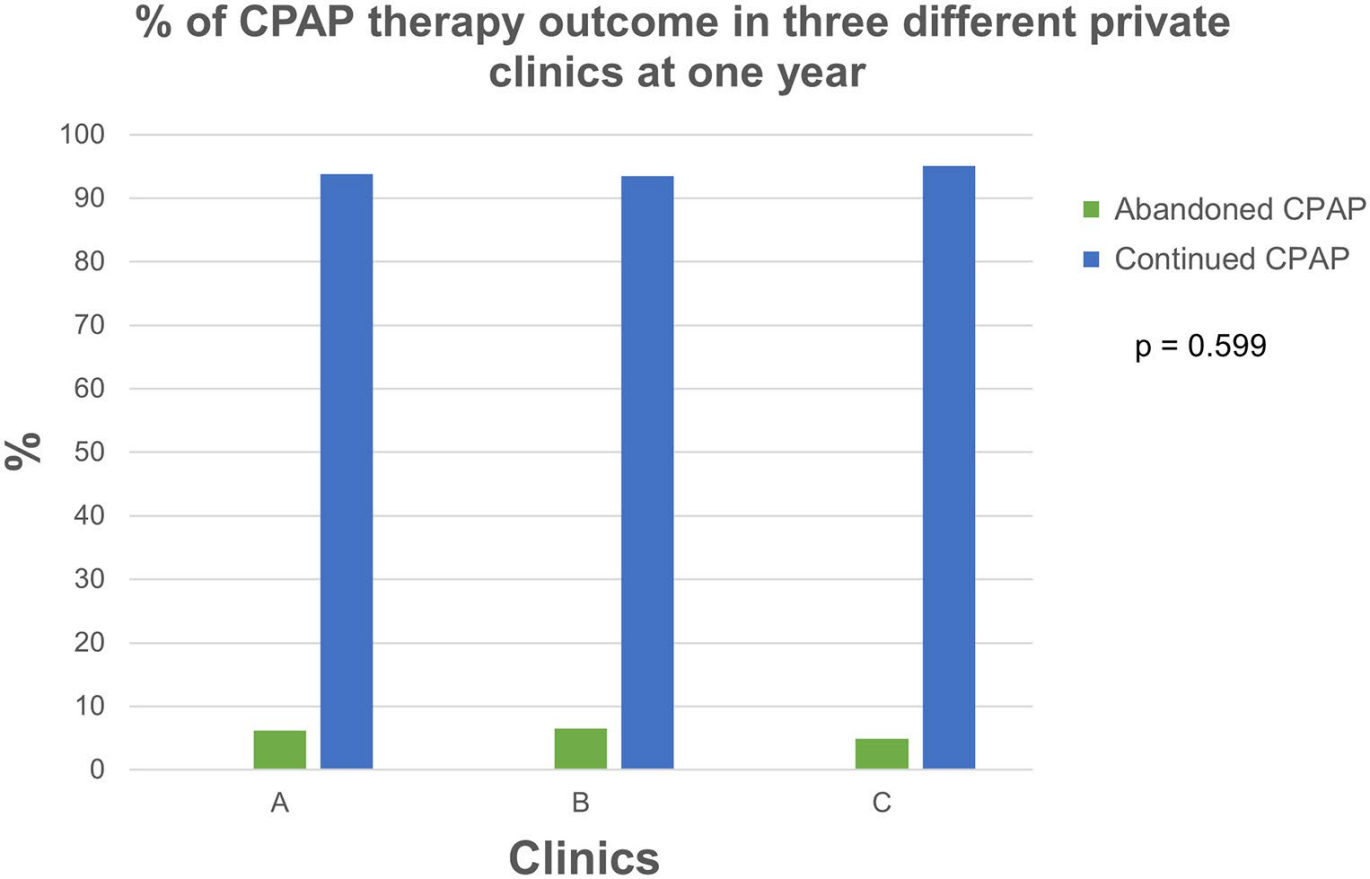
Percentage of patients who continued or abandoned CPAP therapy at one year of initiation

There was no significant (*p* = 0.599) difference between clinics in the number of patients staying on CPAP at 1 year after initiation (Fig. 2).

Significantly more women (8.7%) abandoned CPAP at 1 year than men (4.8%) [χ^2^(1, *N* = 1491) = 9.224, (*p* = 0.003)].

**Fig. 3:**
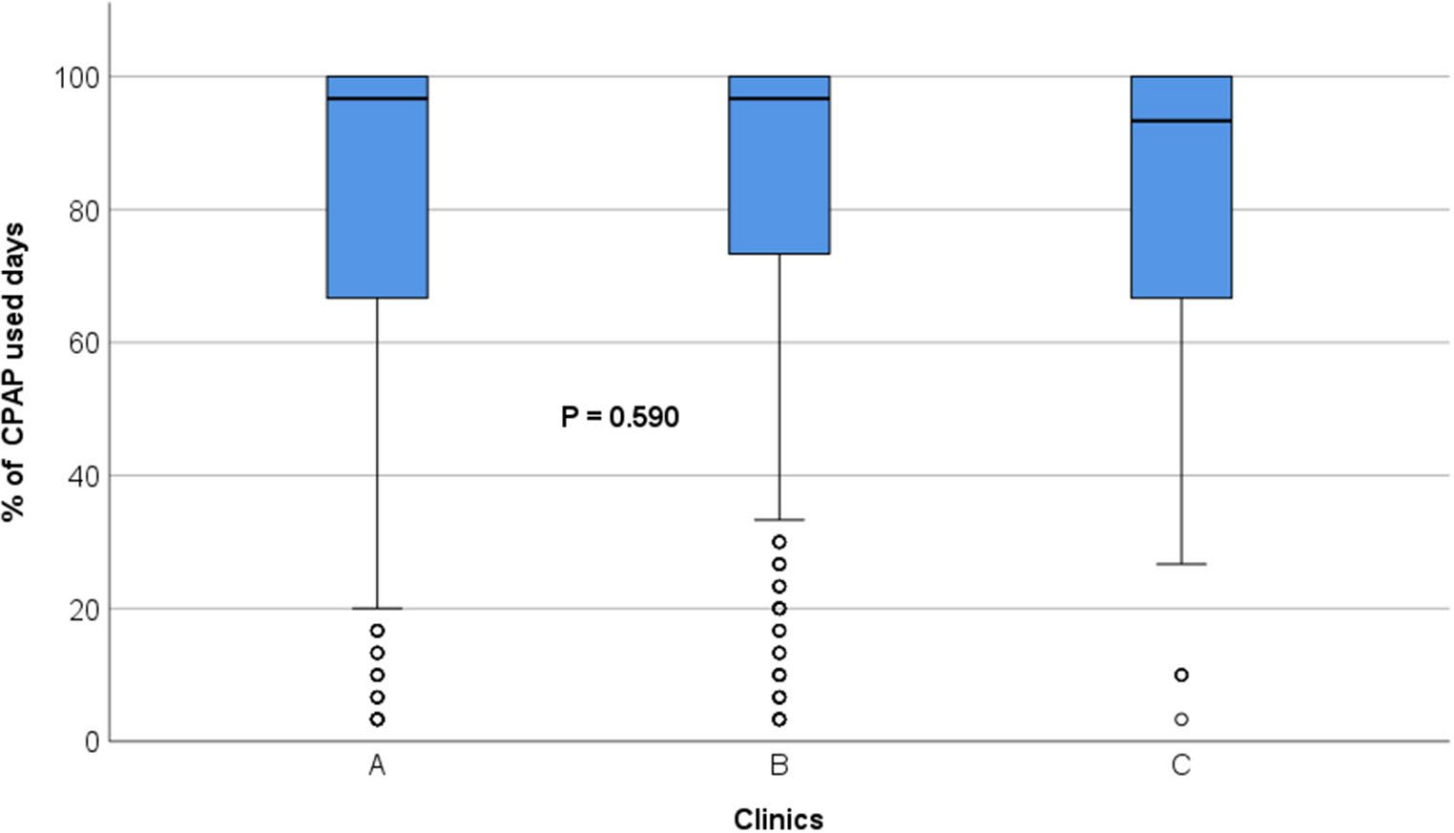
Boxplot showing percentage of days with CPAP use during last visit at 1 year after CPAP initiation. Small circles indicate outliner values

Daily median CPAP use was excellent (97%) at 1 year after initiation. No significant differences were observed in CPAP days used between the three clinics (*p* = 0.590) (Fig. 3).

### CPAP masks and air leaks

**Fig. 4:**
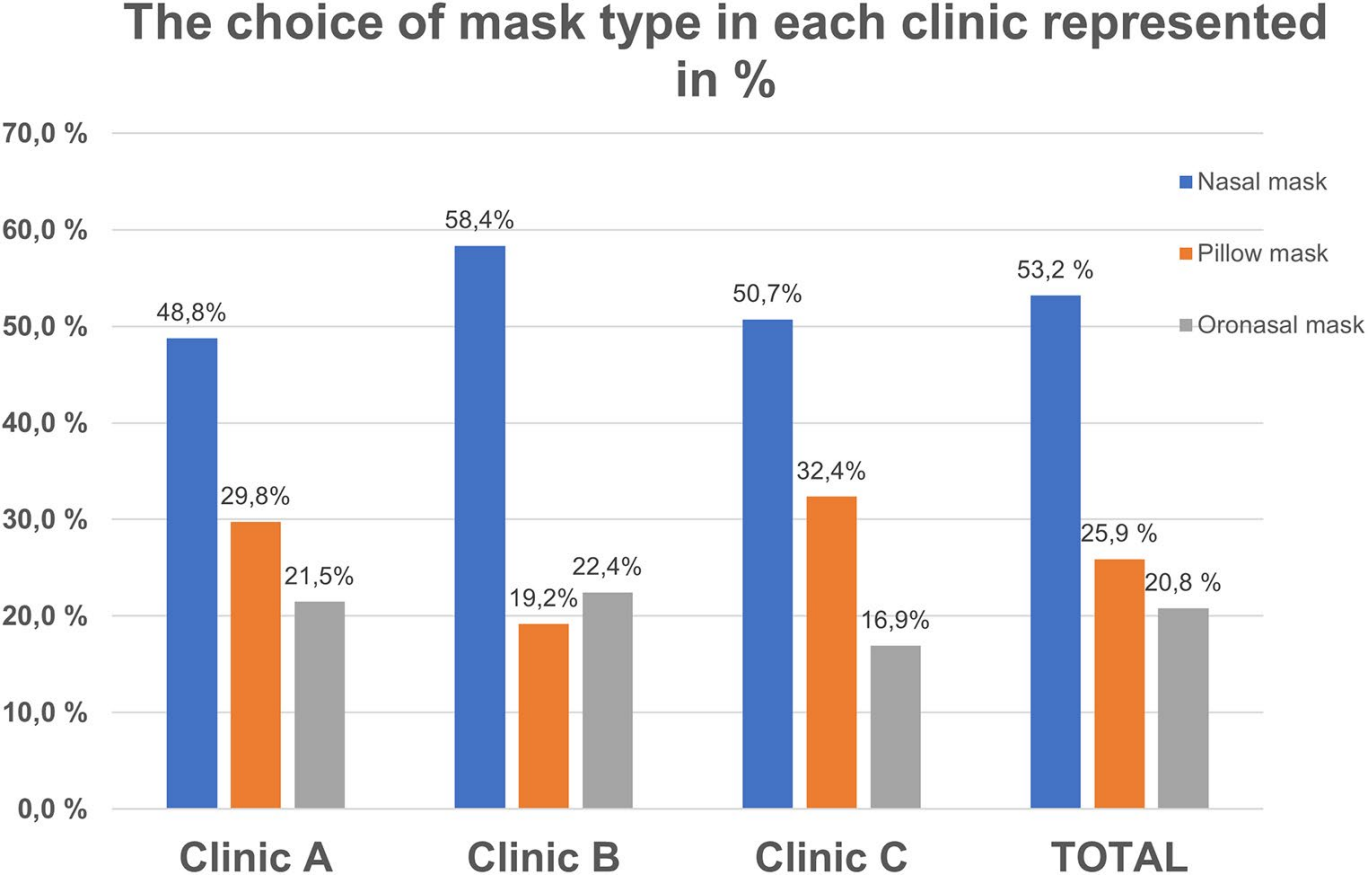
Distribution of the types of masks used in three private clinics

The most frequently used mask type was nasal mask (53%), followed by pillow mask (26%) and oronasal mask (21%). There were no significant differences (*p* > 0.05) in type of mask used between the three clinics (Fig. 4).

The total unintentional air leak was very low (median 3.5 L/min). Leaks were significantly (*p* = 0.011) higher at clinic C (mean [SD] 4.4 [7.0] L/min) than at clinic B (3.6 [5.2] L/min) and clinic A (2.9 [4.3] L/min).

## Discussion

We sent patients from our public sleep apnea clinic to three private clinics for CPAP initiation via voucher use. The success rate of CPAP therapy initiation at 1 year was very high (94%). This is significantly higher than that of our public center, which reported a success rate between 65 and 67% at 1 year [8, 9]. This difference may be related to two significant factors. First, the patients selected for voucher use did not have significant comorbidities, low cognitive capacity, or communication difficulties; these may influence CPAP success rate. Secondly, our criteria for CPAP initiation were recently focused on moderate-to-severe sleep apnea; before 2017 we also treated patients with mild sleep apnea. Balakrishnan et al. reported a significant positive correlation between sleep apnea severity index and CPAP device use, i.e., patients with mild sleep apnea are prone to use their CPAP device significantly less than patients with moderate-to-severe sleep apnea [10]. Moreover, Simon-Tuval T et al. reported that patients with better socioeconomic status had better CPAP outcomes [11]. In Finland, the private sector attracts primarily wealthy patients; in this study, we selected our voucher CPAP patients regardless of socioeconomic status. Moreover, all patients used the same masks and CPAP devices that are used in our public sleep clinic.

There were no significant differences in CPAP therapy success between the three private clinics. This may be due to our strict voucher regulations imposed on these private clinics regarding selection of medical staff, indication of the material used, requirements for reported results, and likely high motivation of these patients as they bypassed the public CPAP therapy waiting list. We previously showed that high willingness score in patients predicts CPAP therapy success at 1 year [8].

We observed that women abandoned CPAP therapy significantly more often than men in this study. Despite being more obese and older, the women in this study had sleep apnea that was slightly less severe than in men. There are several sleep apnea phenotypes and patient gender may affect symptom manifestation of sleep apnea. Bonsignore et al. reported that women appear to have more symptoms with lower AHI scores compared with men [12].

We previously observed that older sleep apnea patients had the same CPAP adherence as younger patients [8]. Although women abandoned CPAP statistically significantly more often than men, the proportions of patients who abandoned CPAP were relatively low.

One of the few studies describing the success of CPAP initiation in private clinics was reported by Lee et al. [13]. The success rate was 53% in this study and their clinic was sponsored. Our success rate was greater this previous study. As our CPAP initiation was performed using a voucher, it is possible that CPAP success could be different if a voucher was not used.

Our study has some limitations. Our conclusion applies to the specific national healthcare insurance system of Finland; therefore, it may not be possible to generalize our results to other healthcare insurance systems. The number of patients was not equal among clinics, but this also reflects the size of each clinic and how it attracts patients.

This study has some strengths. We describe one approach to alleviate the sleep apnea burden on public healthcare systems by engaging private clinics in sleep apnea therapy. The number of included patients is sufficient to draw conclusions. We believe that this study is the first to describe a collaboration between public and private healthcare to address the waiting list for sleep apnea treatment.

## Conclusion

Public clinics are usually overburdened and have restricted budgets. We demonstrated that it is possible to engage the private sector temporarily to perform specific healthcare services, with a relatively high success rate for CPAP therapy initiation as shown here. This success requires clear instructions and supervision of the private sector. When these conditions are applied, we would not expect differences in CPAP outcomes between private clinics, as indicated by our results.

## Data Availability

Data are available on request.
